# Salmon fish protein supplement increases serum vitamin B12 and selenium concentrations: secondary analysis of a randomised controlled trial

**DOI:** 10.1007/s00394-022-02857-4

**Published:** 2022-04-01

**Authors:** Kristin S. Hustad, Inger Ottestad, Thomas Olsen, Thomas Sæther, Stine M. Ulven, Kirsten B. Holven

**Affiliations:** 1 Department of Nutrition, Institute of Basic Medical Sciences, University of Oslo, Oslo, Norway; 2 The Clinical Nutrition Outpatient Clinic, Section of Clinical Nutrition, Department of Clinical Service, Division of Cancer Medicine, Oslo University Hospital, Oslo, Norway; 3 Department of Molecular Medicine, Institute of Basic Medical Sciences, University of Oslo, Oslo, Norway; 4 National Advisory Unit On Familial Hypercholesterolemia, Department of Endocrinology, Morbid Obesity and Preventive Medicine, Oslo University Hospital, Oslo, Norway

**Keywords:** Vitamin B12, Selenium, Sustainability, RCT, Atlantic salmon

## Abstract

**Purpose:**

The main aim of the present study was to examine the effect of a fish protein supplement made from by-products from production of Atlantic salmon, on blood concentration of micronutrients.

**Methods:**

We conducted an 8-week double-blind parallel-group randomised controlled trial. In total, 88 adults were randomised to a salmon fish protein supplement or placebo, and 74 participants were included in the analysis of vitamin D, omega-3, vitamin B12, selenium, folate, zinc, homocysteine and mercury.

**Results:**

During the intervention period, geometric mean (GSD) of serum vitamin B12 concentrations increased from 304 (1.40) to 359 (1.42) pmol/L in the fish protein group (*P* vs. controls = 0.004) and mean (SD) serum selenium increased from 1.18 (0.22) to 1.30 (0.20) μmol/L (*P* vs. controls = 0.002). The prevalence of low vitamin B12 status (B12 < 148–221 > pmol/L) decreased from 15.4 to 2.6% in the fish protein group, while increasing from 5.9 to 17.6% in the placebo group (*P* = 0.045). There was no difference between the groups in serum levels of the other micronutrients measured.

**Conclusion:**

Including a salmon fish protein supplement in the daily diet for 8 weeks, increases serum vitamin B12 and selenium concentrations. From a sustainability perspective, by-products with high contents of micronutrients and low contents of contaminants, could be a valuable dietary supplement or food ingredient in populations with suboptimal intake.

**Trail Registration:**

The study was registered at ClinicalTrials.gov (ID: NCT03764423) on June 29th 2018.

## Introduction

Most dietary guidelines recommend including fish in the diet [1]. The beneficial effects of fish intake have largely been attributed to omega-3 polyunsaturated fatty acids present in fatty fish [2]. However, fish contain other nutrients such as taurine, vitamin D, vitamin B12 (B12), iodine and selenium [3], and intervention studies show that regular consumption of fish increases concentrations of serum vitamin D (s-vitamin D) and B12 (s-B12), urinary iodine and plasma selenium (p-selenium) [4–6]. Worldwide, there are concerns regarding intake and status of several of the micronutrients related to fish intake [7–10].

Norway is one of the world’s largest aquaculture and fishing nations, and the Norwegian fish industry generates a large amount of materials discarded as by-products, mainly utilized for animal feed production [11]. In 2018, only 13% of the by-products were used for human consumption [11]. From a sustainability perspective, it is important to explore available food resources at our disposal. With the expected growth in the aquaculture industry, by-products will become even more available. By-products from fish contain protein, lipids and minerals such as calcium, phosphate, zinc, selenium and iron [12]. Such by-products should ideally be utilized for human consumption [13], e.g. as a novel food ingredient [14].

We have previously reported the effects of eight weeks of daily intake of a fish protein supplement made from by-products from production of Atlantic salmon on cardiometabolic risk markers in adults with increased risk of type 2 diabetes mellitus. We found no beneficial effect on markers related to glucose tolerance, serum lipids, weight or blood pressure compared to the placebo group [15]. In the present secondary analysis, the main aim was to examine the effect on blood concentration of micronutrients related to fish intake or abundant in fish by-products. Additionally, we compared the content of micronutrients and contaminants in the salmon fish protein supplement to the recommended intake (RI) of micronutrients in the Nordic Nutrition Recommendations (NNR) [16] and the tolerable weekly intake (TWI) levels of contaminants set by the European Food Safety Authorities Panel on Contaminants in the Food Chain (EFSA CONTAM Panel) [17–21].

## Methods

### Ethics

The study was conducted according to the guidelines laid down in the Declaration of Helsinki. All participants gave their written informed consent, and the Regional Ethics Committee for Medical Research in South East Norway approved the study (2018/749/REK sør-øst A). The study was registered at ClinicalTrials.gov (ClinicalTrials.gov Identifier: NCT03764423).

### Participants

The present study is based on a previously performed randomised controlled trial, the FishMeal human intervention study, where the primary outcome was changes from baseline in serum glucose (s-glucose) measured after a 2-h oral glucose tolerance test (OGTT). The description of participants, study setting and protocol for study visits have been published in detail previously [15]. In brief, the study was conducted at the University of Oslo, Norway from August 2018 until September 2019. We included weight-stable adults with elevated blood glucose (fasting s-glucose ≥ 5.6 mmol/l, 2 h-OGTT-s-glucose ≥ 6.5 mmol/l or HbA1c ≥ 40 mmol/mol). Exclusion criteria were type 1 and 2 diabetes mellitus, age-related elevated blood pressure, use of glucose lowering drugs, drugs related to inflammation and systemic use of corticosteroids, or unstable use of lipid lowering drugs, thyroxine, blood pressure lowering drugs and drugs affecting appetite. Further exclusion criteria were fish/seafood intake exceeding 450 g/week and allergy to fish or shellfish. Daily users of protein supplement powder, and participants who were pregnant, breastfeeding or planning pregnancy were also excluded. Participants with sporadic/unstable use of dietary supplements were excluded, but participants with regular/stable use of dietary supplement were included.

### Study design

We conducted an 8-week double-blind, randomised controlled parallel trial comparing a salmon fish protein supplement with placebo on blood concentration of micronutrients. Before study start, all participants performed a 2–4-week run-in period with a maximum intake of 150 g of fish and seafood per week. Participants were stratified by sex and age (< 50 y, ≥ 50 y) prior to a block randomisation done by an external statistician. The experimental group received capsules containing salmon fish protein, microcrystalline cellulose, antioxidants and excipients. The placebo group received capsules containing microcrystalline cellulose and antioxidants and excipients similar to the fish protein capsules. We instructed participants in both groups to consume ten capsules together with a meal three times per day for 8 weeks. Capsules were packed in blister sheets with one daily dose per sheet, and delivered in boxes with one weekly dose per box. Participants were instructed to deliver all blister sheets, both empty and full, at the end-of-study visit. Additionally, the participants received a registration scheme to register their intake of capsules. Compliance was assessed by counting the number of capsules consumed during the intervention period, divided by the number of capsules scheduled for the intervention period. All participants had a compliance > 70% [15]. We advised the participants to maintain their usual lifestyle habits throughout the study, including physical activity, supplement use and diet, except for a reduction in fish and seafood intake to a maximum of 150 g per week. Habitual dietary intake was assessed prior to the intervention through a semi-quantitative food frequency questionnaire (FFQ) designed to capture dietary habits during the last year [22]. The same FFQ was used to assess the participants’ diets during the 8-week intervention.

### Study product

The fish protein supplement was made from by-products from production of Atlantic salmon. Content of nutrients and contaminants was analysed before encapsulation (Eurofins Food & Feed Testing Norway AS, Moss, Norway). To evaluate the content of micronutrients, we compared the contribution from a daily dose of capsules to recommended intake (RI) in the Nordic Nutrition Recommendations (NNR) for vitamin D, B12, iodine, selenium, riboflavin, folate, calcium, phosphorous, magnesium, iron and zinc [16]. We used male adults as a reference population. To evaluate the content of contaminants, we compared the contribution from a weekly dose of capsules to the tolerable weekly intake (TWI) levels per kg body weight set by the EFSA CONTAM Panel for arsenic, lead, cadmium, mercury and dioxins and dioxin-like polychlorinated biphenyls (dl-PCBs) [17–21]. We used 70 kg as a reference weight, as recommended by the EFSA Scientific Committee [23]. As no TWI exist for copper, we compared the contribution from a weekly dose of capsules to the tolerable upper intake level (UL) [16].

### Blood sampling and standard laboratory analysis

Venous blood samples were drawn after an overnight fast (≥ 10 h). Participants were instructed to avoid drinking alcohol and doing strenuous physical activity the day before blood sampling. Serum was obtained from silica gel tubes and Lithium Heparin gel tubes (Becton, Dickinson and Company, Franklin Lakes, NJ, USA) and kept at room temperature for > 30–60 < min, until centrifugation (1500×*g*, 15 min). Plasma was obtained from K_2_EDTA tubes (Becton, Dickinson and Company, Franklin Lakes, NJ, USA), immediately placed on ice, and centrifuged within 10 min (2000×*g*, 4 °C, 15 min). Lithium Heparin tubes (Becton, Dickinson and Company, Franklin Lakes, NJ, USA) with whole blood were kept at room temperature. Concentrations of s-B12, s-folate and p-homocysteine were measured on an ADVIA Centaur XPT Immunoassay System analyser (Siemens Healthineers, Tarrytown, NY, USA); s-vitamin D was measured on a Water Acquity UPLC analyser (Waters, Milford, MA, USA); and s-selenium, s-zinc and whole blood mercury were measured on a Nexion 2000 ICP-MS (Perkin Elmer, Shelton, CT, USA) by standard methods at an accredited routine laboratory (Fürst Medical Laboratory, Oslo, Norway). P-omega-3 was measured using a high-throughput nuclear magnetic resonance spectroscopy platform (Nightingale Health, Helsinki, Finland).

### Statistical analysis

Power calculation was originally conducted with the aim to investigate the effect on s-glucose 2 h after an OGTT. We estimated that 120 participants (including a 20% dropout rate) were required to obtain 80% power with a type I error of 5% to detect a clinically relevant difference between the two groups of 0.4 mmol/l (standard deviation (SD) 0.7).

Data are presented as mean (SD) or median (quartiles, Q1–Q3) for continuous variables or as frequency (percentage, %) for categorical variables. Differences between the groups were tested with a linear regression model (outcome variable ~ intervention group + outcome variable at baseline), hereafter called crude model. We performed the same analysis adjusting for strata (age and sex) in addition to the outcome variable at baseline (outcome variable ~ intervention group + outcome variable at baseline + age + sex), hereafter called the adjusted model. Skewed variables (s-B12, s-folate, p-omega-3 and whole blood mercury) were log-transformed before analysis. Results from the regression analysis are presented as β-coefficient with 95% confidence interval (CI) or logβ-coefficient with 95% CI for skewed variables (s-B12, s-folate, p-omega-3 and whole blood mercury). Baseline and end-of-study values for skewed variables are presented as geometric mean and geometric standard deviation (GSD). *P* < 0.05 was considered significant. The models were checked for independence and normality of the residuals. Differences in B12 status (low B12 status (B12 < 148–221 > pmol/L) and B12 deficiency (B12 < 148 pmol/L)) were tested using two-sided Fisher’s exact test. One participant was excluded from the analysis of s-B12, p-homocysteine and B12 status due to B12-injection during the intervention period.

Statistical analysis were performed in Stata/MP 16.0 (StataCorp LLC, College Station, TX, USA) [24] and the figure was created in R (R Foundation for Statistical Computing, Vienna, Austria) [25].

## Results

In total, 717 participants were assessed for eligibility, 88 were randomly assigned, 83 received allocated interventions and 7 were lost to follow-up. Thus, 76 participants completed the study. Two participants were non-compliant with the study protocol, leaving a total of 74 participants included in the statistical analysis, as previously reported [15]. The participants were 56 (Q1–Q3: 48–64) years of age, with a BMI of 33.1 (Q1–Q3: 29.9–36.1) kg/m^2^ and 64% were female. Further participant characteristics and information on dietary intake is summarized in Table 1.

**Table 1:** Participant characteristics (*n* = 74) and dietary intake at baseline

	*n* = 74
	Median (Q1–Q3)
Age, years	56 (48–64)
Sex, female, n (%)	47 (64)
BMI, kg/m^2^	33.1 (29.9–36.1)
hsCRP, mg/L	3.4 (2.1–6.0)
Daily tobacco use, n (%)	11 (15)
Energy, kJ/d	9260 (7940–11399)
Protein, E%	16.7 (15.4–18.6)
Fat, E%	35.5 (32.8–39.7)
Carbohydrates, E%	40.6 (35.9–45.9)
Alcohol, E%	1.9 (0.5–6.2)

Median values and quartiles (Q1–Q3); frequencies and percentages (%)

*BMI* body mass index, *hsCRP* high-sensitive C-reactive protein, *kJ* kilojoule, *E%* percentage of total energy intake

### Content of micronutrients and contaminants in the fish protein supplement

Content of nutrients and contaminants in the fish protein supplement is summarized in Tables 2 and 3. One daily dose of the fish protein supplement provided 4.5 μg B12, corresponding to 227% of the RI (Table 2). For other micronutrients measured in the study product, the content corresponded to 117% of the RI for zinc, 29% for selenium, 26% for phosphorous, 23% for calcium, 22% for riboflavin, 15% for folate, 9% for iron, 9% for iodine, 4% for vitamin D and 3% for magnesium.

**Table 2:** Content of nutrients in the salmon fish protein supplement

	Salmon fish protein supplement, per 100 g	Salmon fish protein supplement, per 7.5 g (daily dose)	Recommended intake per day^a^	% contribution of recommended intake per 7.5 g (daily dose)	Upper limit^b^
Micronutrients					
Vitamin D (μg)	4.8	0.4	10	4	100
Vitamin B12 (μg)	60.5	4.5	2	227	NE
Iodine (μg)	170	12.8	150	9	600
Selenium (μg)	230	17.3	60	29	300
Riboflavin (mg)	5.1	0.4	1.7	22	NE
Folate (μg)	599	44.9	300	15	NE
Calcium (mg)	2500	187.5	800	23	2500
Phosphorous (mg)	2100	157.5	600	26	3000
Magnesium (mg)	140	10.5	350	3	NE
Iron (mg)	11	0.8	9	9	60
Zinc (mg)	140	10.5	9	117	NE
Macronutrients					
Total protein (g)	69.7	5.2			
Total fat (g)	13.2	1.0			
Sum EPA + DHA (mg)	2020	151			

*NE* not established

^a^Values from Nordic Nutrition Recommendations 2012, reference population: adult male, 31–60 years

^b^Values from Nordic Nutrition Recommendations 2012

**Table 3:** Content of contaminants in the salmon fish protein supplement

	Salmon fish protein supplement	Tolerable weekly intake^a^	Tolerable weekly intake for adult 70 kg	Weekly intake from salmon protein supplement	% contribution of tolerable weekly intake per week
Heavy metals					
Arsenic (mg/kg)	1.60	15 μg/kg	1050 μg	84 μg	8
Lead (mg/kg)	< 0.05	25 μg/kg	1750 μg	2.6 μg^b^	0.1
Cadmium (mg/kg)	0.08	2.5 μg/kg	175 μg	4.2 μg	3
Copper (mg/kg)	86	5 mg/d	35 mg	4.5 mg	13
Mercury (mg/kg)	0.11	4 μg/kg	280 μg	5.8 μg	2
Organic pollutants^c^					
Dioxins (ng/kg)	0.08				
Dioxin-like PCBs (ng/kg)	0.10				
Dioxins and dl-PCBs (ng/kg)	0.18	2 pg/kg	140 pg	9.4 pg	7
PCB 6 (μg/kg)	1.27				
PCB 7 (μg/kg)	1.40				

*PCBs* polychlorinated biphenyls, *dl-PCBs* dioxin-like polychlorinated biphenyls

^a^Tolerable weekly intakes (TWI) from EFSAs CONTAM Panel. No longer appropriate for arsenic (2009) and lead (2013). For copper: tolerable upper intake level from NNR. TWI for dioxins and dl-PCBs will protect against too high intakes of PCB 6 and PCB 7

^b^Content of lead in salmon fish protein supplement used for calculation of weekly intake: 0.05

^c^Reported concentrations of organic pollutants are upper-bound

Weekly intake of capsules did not exceed UL or TWI for any of the examined contaminants in the supplement for a person with a body weight of 70 kg (Table 3).

### Effect of fish protein supplementation on blood concentrations of nutrients

**Fig. 1:**
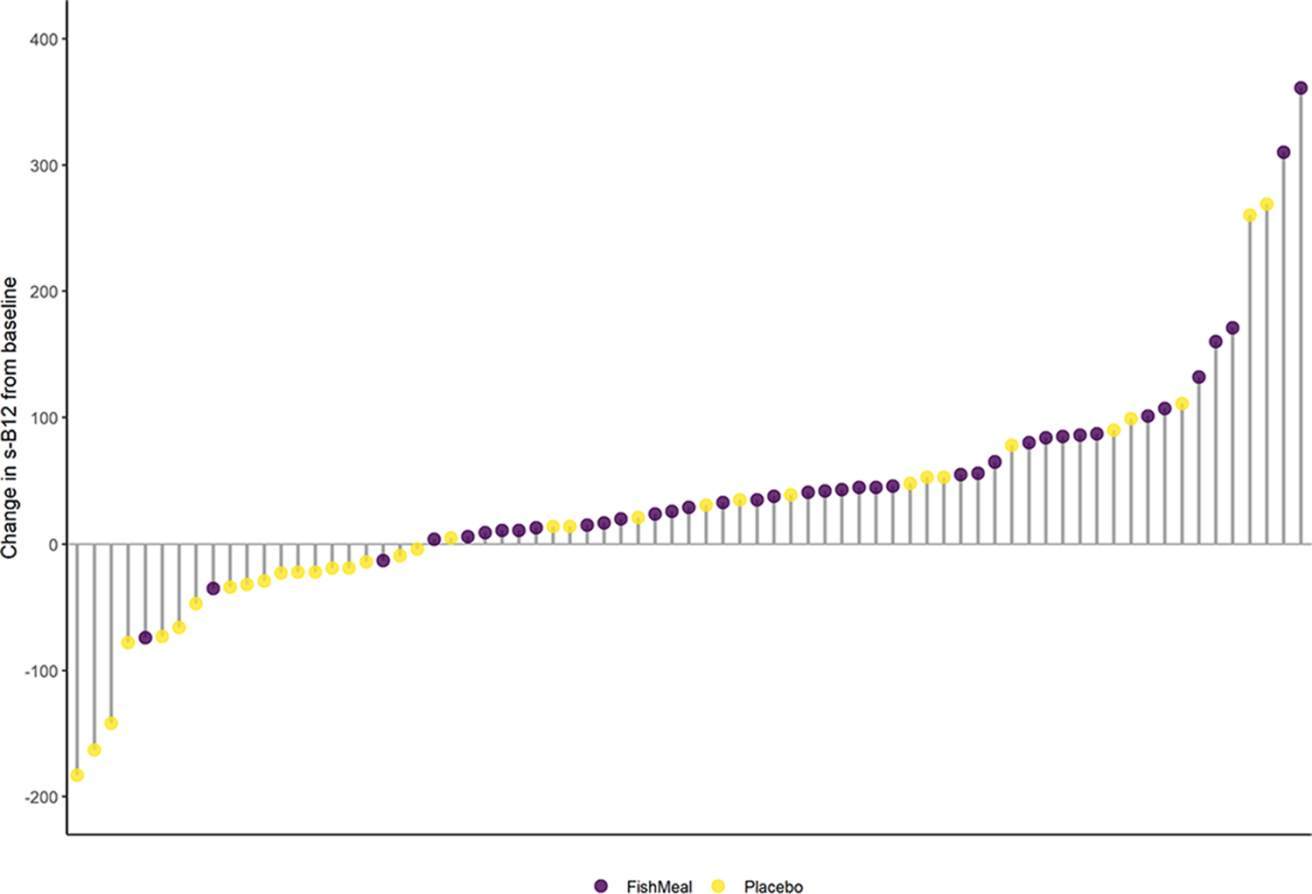
Individual changes in s-B12 from baseline

Differences between the fish protein group and the placebo group on blood concentrations of nutrients using crude and adjusted models are shown in Table 4. During the 8-week intervention period, geometric mean (GSD) of s-B12 concentrations increased from 304 (1.40) to 359 (1.42) pmol/L in the fish protein group (*P* vs. controls = 0.004) in the adjusted analysis. Individual responses on s-B12 concentration is illustrated in Fig. 1.

**Table 4:** Effect of 8 weeks of salmon fish protein supplementation on blood concentrations of nutrients

	N	Fish protein		Placebo		Change in the fish protein group relative to the placebo group			
		*n* = 39		*n* = 35					
		Baseline	End-of-study	Baseline	End-of-study				
		Mean (SD)	Mean (SD)	Mean (SD)	Mean (SD)	Crude values B (95% CI)	Adjusted values B (95% CI)	^*a*^*P*	^b^*P*
Nutrient									
Vitamin B12 (pmol/L)^c^	73	304 (1.40)	359 (1.42)	321 (1.33)	325 (1.38)	0.15 (0.05–0.25)	0.14 (0.05–0.24)	0.004	0.004
Selenium (μmol/L)	73	1.18 (0.22)	1.30 (0.20)	1.21 (0.29)	1.21 (0.17)	0.10 (0.04–0.15)	0.10 (0.04–0.16)	0.002	0.002
Folate (nmol/L)^c^	71	14.5 (1.58)	15.1 (1.62)	15.2 (1.59)	14.7 (1.50)	0.05 (− 0.07–0.17)	0.05 (− 0.07–0.16)	0.382	0.411
Vitamin D (nmol/L)	74	63 (20)	61 (18)	68 (21)	66 (20)	− 0.92 (− 6.26–4.42)	− 0.90 (− 6.15–4.35)	0.731	0.734
Omega-3 (mmol/L) ^c^	74	0.54 (1.41)	0.55 (1.39)	0.57 (1.30)	0.56 (1.34)	0.03 (− 0.05–0.12)	0.03 (− 0.06–0.12)	0.455	0.462
Zinc (μmol/L)	67	13.7 (1.5)	13.4 (1.3)	13.7 (1.6)	13.6 (1.5)	− 0.27 (− 0.84–0.31)	− 0.27 (− 0.84–0.31)	0.359	0.358
Miscellaneous									
Homocysteine (μmol/L)	72	10.2 (2.9)	9.3 (2.8)	10.4 (2.5)	10.0 (2.2)	− 0.58 (− 1.35–0.19)	− 0.56 (− 1.35–0.22)	0.140	0.158
Mercury (nmol/L)^c^	70	6.6 (1.47)	6.1 (1.38)	7.8 (1.59)	6.7 (1.51)	0.01 (− 0.10–0.11)	0.01 (− 0.10–0.11)	0.913	0.895

Differences between the groups were tested with a linear regression model. The regression coefficient expresses the mean difference between the groups. A negative regression coefficient in this table represents a reduction in the fish protein group compared to the placebo group, and a positive regression coefficient represents an increase

Mean values and standard deviations (SD); B-coefficients and 95% confidence intervals (95% CI)

^a^*P* for difference between the fish protein group and placebo group using crude values: end-of-study values adjusted for group and baseline values

^b^*P* for difference between the fish protein group and placebo group using adjusted values: end-of-study values adjusted for group, baseline values, age and sex

^c^Skewed variables (vitamin B12, folate, omega-3 and mercury) were log-transformed before analysis and presented as geometric mean (GSD), exp(logB) coefficients and 95% CI

Since we found a significant increase in s-B12 in the fish protein group vs. the placebo group, we investigated whether the supplement could affect B12 status based on established clinical cut-offs. The results are summarized in Table 5. At baseline, there were no difference between the groups in prevalence of low B12 status (B12 < 148–221 > pmol/L) (*P* = 0.271). At the end-of-study, 2.6% of the participants in the fish protein group and 17.6% in the placebo group had low B12 status (*P* vs. controls = 0.045). None of the participants had B12 deficiency (B12 < 148 pmol/L) at either time point.

**Table 5:** Prevalence of low B12 status at baseline and after 8 weeks of salmon fish protein supplementation

	Baseline	End-of-study	^a^*P*	^b^*P*
Low B12 status (< 148–221 > pmol/L)			0.271	0.045
Fish protein	6 (15.4)	1 (2.6)		
Placebo	2 (5.9)	6 (17.6)		

Differences between the groups were tested with 2-sided Fisher’s exact test

None of the participants had B12 deficiency (< 148 pmol/L) at either time point

Frequency and percentage (%)

^a^*P* Difference between the groups at baseline.

^b^*P* Difference between the groups at end-of-study

During the intervention period, mean (SD) s-selenium increased from 1.18 (0.22) to 1.30 (0.20) μmol/L in the fish protein group (*P* vs. controls = 0.002). S-folate, s-vitamin D, p-omega-3, s-zinc, p-homocysteine or whole blood mercury did not change during the intervention period.

## Discussion

In the present study, we examined the effect of including a fish protein supplement made from by-products from production of Atlantic salmon in the daily diet for 8 weeks, on blood concentration of micronutrients. We found that 8 weeks of fish protein supplementation, which was a good source of micronutrients and had a low content of contaminants, increased serum concentrations of B12 and selenium among adults. To the best of our knowledge, this is the first clinical trial examining the effects of a fatty fish protein supplement on blood concentration of micronutrients.

Although the study product in the present study was made from fatty fish, we did not find an effect on vitamin D and omega-3, despite omega-3 being a well-established biomarker of fatty fish intake [26]. P-omega-3 increases in a dose-dependent manner, however, most studies examining this relationship have provided higher doses of EPA and/or DHA than the study product, providing 151 mg/d of EPA and DHA [27]. Additionally, about one third of the participants in the present study used omega-3 supplements regularly prior to the study and during the intervention (data not shown). As the daily dose of the study product provided only 0.4 μg vitamin D, we did not expect an increase in s-vitamin D. Additionally, since many were using supplements with vitamin D regularly (data not shown), we did not expect a decrease in s-vitamin D. In an 8-week RCT in adults (*n* = 63) with overweight or obesity comparing intake of salmon or cod consumed as whole filet, 750 g/week of salmon was not sufficient to prevent a decrease in s-vitamin D in autumn in South-Western Norway, despite a median vitamin D intake of 11.9 μg/d [28], which was above the recommendation of 10 µg/d [16].

Our findings on s-B12 and s-selenium are in line with intervention studies on intake of fish consumed as whole filet. A randomised cross-over study in overweight men (*n* = 32) comparing herring with chicken and pork (750 g/week for 6 weeks) found an increase in s-B12 (8.9%) and s-selenium (4.6%), but not s-vitamin D, during the herring diet [5]. In the previously mentioned RCT comparing intake of salmon or cod consumed as whole filet (750 g/week), only cod increased s-selenium compared to controls [29]. We have previously performed a post-prandial analysis of serum amino acids in five healthy adults after intake of the study product, and found a non-significant increase in serum levels of most amino acids [15], supporting that the capsules were absorbed. Both B12 and selenium have high bioavailability and serum and plasma concentrations reflects dietary exposure [7, 30], supporting our findings in the present study.

In line with our findings on increased s-B12 concentration, participants in the salmon fish protein group, but not in the placebo group, improved their B12 status during the intervention period. Although we did not detect any participants with B12 deficiency in the present study, 11% had low B12 status at baseline in the two groups combined. However, although s-B12 is a useful indicator of B12 status, it lacks sensitivity and specificity for diagnosing B12 deficiency [7].

Although the worldwide prevalence of B12 deficiency is uncertain, it is estimated to exceed 40% in subpopulations such as children, women of childbearing age and older adults in low- and middle-income countries [7]. In Europe, existing prevalence data on B12 deficiency mostly focus on the elderly, and varies from 5.9% in a Norwegian study [31] to 24% in a study from the Netherlands [32]. Less is known about selenium status. However, low selenium intake is reported in several European countries, as selenium content in food is dependent on contents in the soil the food is produced and the selenium contents of European soil is generally low [33]. It is estimated that 40% of pregnant women in the Norwegian mother and child cohort had an inadequate selenium intake [34].

In a study on preschool children (*n* = 210), consumption of fatty fish three times per week for 16 weeks resulted in higher concentrations of hair mercury compared to meat [35], even though the exposure for fish mercury from the intervention did not exceed the TWI. Similarly, the contribution from a weekly dose of capsules in the present study did not exceed the TWI. Benefit-risk assessments of seafood conclude that the benefits outweigh the potential harmful effects from contaminants [36, 37]. Both selenium and zinc were abundant in the study product in the present study, and have the potential to antagonize toxic effects of heavy metals [38].

The strengths of the present fish protein human intervention study are the randomised controlled double-blind design, while the main limitation is that power calculation was done for the primary endpoint in the original study, and not the outcomes in the present study. To avoid overestimation of the contribution of micronutrients to the RI from a daily dose of capsules, we used male adult as a reference population, as RI for men are the same or higher as those for women, except from iron. Additionally, RI for several micronutrients is higher during pregnancy and breastfeeding [16]. Thus, another limitation is that different assessments must be done to evaluate the potential use of the study product in other populations.

Low food intake in general or low intake of foods of animal origin, may cause low intake of several nutrients abundant in the study product, such as vitamin B12, zinc and selenium. To ensure sufficient intake of these nutrients in such populations, one possibility is to use by-products from fish as a dietary supplement or food ingredient. A study examining the sensory acceptance of bread baked with added fish flour, found that the acceptance was better than, or as good as, the bread without fish flour. Additionally, the bread with fish flour had higher contents of protein, essential fatty acids, and minerals, but lower content of carbohydrates [39].

In conclusion, in the present study, a fish protein supplement made from by-products from production of Atlantic salmon included in the daily diet for 8 weeks, increased serum vitamin B12 and selenium concentrations. From a sustainability perspective, by-products with high contents of micronutrients and low contents of contaminants, as in the present study, could be a valuable dietary supplement or food ingredient in populations with suboptimal intake. The potential use of by-products from the aquaculture and fishery industry for human consumption should, therefore, be further elucidated.

## Data Availability

Anonymised data used in the manuscript will be available upon request.

## Code availability

Stata/MP 16.0 (StataCorp LLC, College Station, TX, USA).
